# The physical, mental, and social impact of COPD in a population-based sample: results from the Longitudinal Aging Study Amsterdam

**DOI:** 10.1038/s41533-018-0097-3

**Published:** 2018-08-10

**Authors:** Frits M. E. Franssen, Dionne E. Smid, Dorly J. H. Deeg, Martijn Huisman, Jan Poppelaars, Emiel F. M. Wouters, Martijn A. Spruit

**Affiliations:** 1 Department of Research & Education, CIRO, Horn, The Netherlands; 2 NUTRIM School of Nutrition and Translational Research in Metabolism, Maastricht, The Netherlands; 3 Department of Respiratory Medicine, Maastricht University Medical Centre, Maastricht, The Netherlands; 4 Department of Epidemiology & Biostatistics, EMGO+Institute for Health and Care Research, VU University Medical Center, Amsterdam, The Netherlands; 5 Department of Sociology, VU University, Amsterdam, The Netherlands; 6 REVAL - Rehabilitation Research Center, BIOMED - Biomedical Research Institute, Faculty of Medicine and Life Sciences, Hasselt University, Diepenbeek, Belgium

## Abstract

Chronic obstructive pulmonary disease (COPD) is associated with substantial health impact that may already become apparent in early disease. This study aims to examine the features of subjects with COPD in a Dutch population-based sample and compare their physical status, mental status, and social status to non-COPD subjects. This study made use of Longitudinal Aging Study Amsterdam (LASA) data. Demographics, clinical characteristics, self-reported diseases, post-bronchodilator spirometry, physical, mental, and social status were assessed. A number of 810 subjects (50.5% male, mean age 60.5 ± 2.9 years) were included. Subjects with COPD (*n* = 68, mean FEV_1_ 67.6 [IQR 60.4–80.4] %.) had a slower walking speed than non-COPD subjects, *p* = 0.033. When compared to non-COPD subjects, COPD subjects gave a lower rating on their health (physical subscale of SF-12: 15 [IQR 16.0–19.0] vs. 18 [IQR 11.0–17.0] points) and life (EQ5D VAS: 75 [IQR 70.0–90.0] vs. 80 points [IQR 65.0–85.5]) surveys. COPD subjects also had a more impaired disease-specific health status (CAT: 9.5 ± 5.9 vs. 6.7 ± 5.2, respectively), were less likely to have a partner (69% vs. 84%, respectively) and received emotional support less often (24% vs. 36%, respectively) compared to non-COPD subjects (All comparisons *p* < 0.001). In a population-based sample, subjects with COPD had a reduced physical performance, a more impaired disease-specific health status and were more socially deprived compared to non-COPD subjects. These impairments need to be taken into consideration when setting up a management program for patients with mild COPD.

## Chronic lung disease: Comprehensive support from the early stages

Patients with early-stage chronic lung disease need holistic support to limit the physical, mental and social impacts of the condition. There is more to chronic obstructive pulmonary disease (COPD) than persistent airflow limitation; systemic effects, including loss of muscle strength and higher risk of heart conditions, mental health and social problems can manifest from the early stages. Frits Franssen at CIRO, the Netherlands, and co-workers interviewed 810 participants aged 55–65 from the Longitudinal Aging Study Amsterdam to investigate the physical, mental and social status of COPD sufferers and compare them with healthy controls. Those with COPD were more likely to walk slower, tire easily and perceive themselves as having poor overall health. Socially, COPD patients were less likely to have long-term partners and felt the need for more emotional support than their healthy peers.

## Introduction

Chronic obstructive pulmonary disease (COPD) is a major cause of morbidity and mortality around the world.^1^ It is well recognized that the burden of disease for the individual patient is only partially reflected by the degree of airflow limitation.^2^ Most studies aimed at investigating the burden of disease were performed in selected populations of diagnosed patients.^3,4^ Subjects with a less significant smoking history (≤15 pack years), comorbidities with COPD-like symptoms, less frequent exacerbations, and a better lung function were often excluded from these studies.^5^ In addition, subjects with COPD visiting a chest physician or referred for pulmonary rehabilitation were overrepresented.^3,5^ While undiagnosed subjects seem to be healthier than subjects with a diagnosis of obstructive lung disease, studies have shown that these subjects have an impaired health and functional status, and increased risk of death compared to non-COPD subjects.^6,7^ This highlights the importance of increasing our understanding of COPD in a broader population without exclusion of patients with mild to moderate COPD, a less pronounced smoking history and/or specific comorbidities in order to improve the generalizability and clinical significance of findings.

COPD is nowadays recognized as a multicomponent disease, despite being defined by the presence of persistent airflow limitation. The disease also affects systems and organs outside the lungs, the so-called systemic effects of COPD (e.g., weight loss, muscle dysfunction, cardiovascular disease).^8^ For instance, previous research indicated that subjects with COPD have a lower physical activity level, even early in the disease process,^9^ a substantially impaired lower limb muscle and handgrip strength,^10^ and a lower exercise capacity in comparison with non-COPD subjects.^11^ Subjects with COPD also have a worse mental status compared to non-COPD subjects (e.g., more symptoms of anxiety and depression), a lower quality of life, more cognitive dysfunction and more symptoms of fatigue.^12–14^ While it was shown that the diagnosis of COPD has social consequences,^15,16^ little is known about how they manifest in daily living (e.g., personal network size, the frequency of daily support or satisfaction with received help). The combination of the pulmonary abnormalities and these systemic effects of COPD determines the integrated health status.^17^ Therefore, the current study had the following aims: (1) to study the features (e.g., age, gender, smoking history, lung function, comorbidities) of subjects with COPD in a Dutch population-based sample and (2) to compare physical, mental, and social status in this sample with a non-COPD sample from the same population.

## Results

In total, 889 subjects were included in the measurement wave in 2012/13. Of those, 810 subjects completed spirometry (see appendix Figure E1 for flow-chart), of which 742 subjects (91.6%) had a FEV_1_/FVC above the 5th percentile and 68 subjects (8.4%) fulfilled the diagnostic criterion for chronic airflow limitation; 18 GOLD grade 1; 43 GOLD grade 2; 5 GOLD grade 3; and 2 GOLD grade 4. Of these, 68 subjects with COPD, 27 subjects (40%) were previously diagnosed with a respiratory disease (self-reported).

### Features of the COPD subjects

Features of the study subjects are presented in Table 1. Subjects with and without COPD were comparable in terms of age, gender distribution, BMI and blood pressure. While previous cardiovascular comorbidities and diabetes were more frequently present in subjects with COPD than in those without, statistical significance was not reached. Notably, a high percentage of incontinence was found in both groups (non-COPD subjects: 15.9%, and COPD subjects: 14.7%). COPD subjects were more likely to be current smokers, had more pack-years and used more medications than non-COPD subjects.

**Table 1:** Characteristics of study subjects

	Non-COPD subjects	COPD subjects
*N*	742	68
Men, *n* (%)	372 (50.1)	36 (52.9)
Age, years	60.4 (2.9)	60.9 (2.8)
Current smoker, *n* (%)	120 (16.2)^*^	28 (41.2)
Packyears, *n*	3.9 (0.0–18.8)^*^	23.6 (10.2–41.1)
FEV_1_,% predicted	99.5 (90.4–109.6)^*^	67.6 (60.4–80.4)
FEV_1_/FVC, %	79.9 (75.7–83.2)^*^	61.6 (54.2–64.3)
BMI, kg/m^2^	26.6 (23.8–29.5)	26.0 (23.3–28.9)
Systolic blood pressure, mm Hg	136.0 (123.0–149.0)	134.0 (125.0–149.8)
Diastolic blood pressure, mm Hg	83.0 (75.0–90.0)	81.5 (75.0–91.0)
Number of SR comorbidities, *n*	1.5 (1.4)	1.7 (1.4)
Respiratory diseases, *n* (%)	46 (6.2)^*^	27 (39.7)
Heart diseases, *n* (%)	77 (10.4)	12 (17.6)
Peripheral artery diseases, *n* (%)	22 (3.0)	4 (9.8)
Diabetes, *n* (%)	57 (7.7)	5 (12.2)
CVA, *n* (%)	16 (2.2)	2 (4.9)
Incontinence, *n* (%)	118 (15.9)	10 (14.7)
Osteoarthritis, *n* (%)	307 (41.4)	24 (35.3)
Rheumatoid arthritis, *n* (%)	59 (8.0)	5 (7.4)
Cancer, *n* (%)	67 (9.0)	7 (10.3)
Other chronic diseases, *n* (%)	248 (33.4)	17 (25.0)
Number of medications, *n*	1.0 (0.0–2.0)^*^	2.0 (0.0–4.8)

Values expressed as mean (SD), median (IQR), or number of patients (%)

*FEV*_*1*_ forced expiratory volume in the first second, *FVC* forced vital capacity, *BMI* body mass index, *FFMI* fat-free mass index, *SR* self-reported

^*^p ≤ 0.05 vs. group COPD patients

### Physical status

COPD subjects rated their physical health worse (*p* < 0.001) and walked more slowly on a distance of 6 meters (*p* = 0.033) compared to non-COPD subjects, see Table 2. The number of falls in the last year, the experience of pain, sleep quality, self-reported sedentary behavior, handgrip strength, and use of aids in daily life were comparable between groups. Noteworthy was the high percentage of subjects who experienced pain (non-COPD subjects: 27.4%, and COPD subjects: 31.3%) in both groups.

**Table 2:** Physical status of the study subjects divided in patients with and without COPD

SR health		
Poor, *n* (%)	13 (1.8)	2 (2.9)
Sometimes good, *n* (%)	72 (9.7)^*^	13 (19.1)
Fair, *n* (%)	125 (16.8)	15 (22.1)
Good, *n* (%)	406 (54.7)	31 (45.6)
Excellent, *n* (%)	126 (17.0)	7 (10.3)
SF-12 physical health, points	18.0 (16.0–19.0)^*^	15.0 (11.0–17.0)
Number of falls last year	1.0 (1.0–2.0)	1.0 (1.0–2.3)
Experience pain during the day, *n* (%)	189 (27.4)	20 (31.3)
Sleep quality		
Very bad, *n* (%)	25 (3.6)	4 (6.2)
Somewhat bad, *n* (%)	88 (12.6)	9 (13.8)
Somewhat good, *n* (%)	294 (42.1)	26 (40.0)
Very good, *n* (%)	283 (38.1)	26 (40.0)
No sleeping problems, *n* (%)	97 (13.9)	8 (12.3)
SR sedentary behavior, minutes	775.0 (570.0–1020.0)	780.0 (600.0–1140.0)
Handgrip strength right, kg/force	32.7 (13.0)	33.4 (11.6)
Handgrip strength left, kg/force	32.3 (12.9)	32.5 (12.7)
6 m walk test, seconds	6.0 (2.8)^*^	6.5 (2.5)
Use of aids in daily life, *n* (%)	17 (2.3)	3 (4.4)

Values expressed as mean (SD), median (IQR), or number of patients (%)

SR self-reported

^*^p ≤ 0.05 vs. group COPD patients

### Mental status

A large proportion of subjects with COPD had neither a positive nor a negative view of their overall life. However, COPD subjects had a more impaired disease-specific health status and rated their health less positive than non-COPD subjects (Table 3). Cognitive status, depressive symptoms, symptoms of anxiety, generic health status, and mental status were comparable between groups.

**Table 3:** Mental status of the study subjects divided in patients with and without COPD

	Non-COPD subjects	COPD subjects
15WT		
Learning memory, points	23.0 (19.0–27.0)	22.5 (18.0–26.0)
First retention, points	75.0 (60.0–88.9)	72.7 (54.3–85.7)
Second retention, points	72.7 (60.0–86.7)	73.9 (58.5–85.4)
CES-D, points	13.0 (11.0–15.0)	13.0 (11.0–16.0)
HADS-A, points	4.0 (3.0–5.0)	4.0 (3.0–5.0)
Life satisfaction		
Very dissatisfied, n (%)	3 (0.4)	1 (1.5)
Dissatisfied, n (%)	16 (2.2)	2 (2.9)
Neutral, n (%)	91 (12.3)^*^	14 (20.6)
Satisfied, n (%)	472 (67.5)	41 (60.3)
Very satisfied, n (%)	109 (15.6)	6 (8.8)
CAT, points	6.7 (5.2)^*^	9.5 (5.9)
EQ5D		
Mobility, points	1.0 (1.0–1.0)	1.0 (1.0–2.0)
Self-care, points	1.0 (1.0–1.0)	1.0 (1.0–1.0)
Usual activities, points	1.0 (1.0–1.0)	1.0 (1.0–2.0)
Pain/discomfort, points	1.0 (1.0–1.0)	1.0 (1.0–2.0)
Anxiety/depression, points	1.0 (1.0–1.0)	1.0 (1.0–1.0)
VAS, points	80.0 (70.0–90.0)^*^	75.0 (65.0–85.5)
SF-12 mental health, points	23.0 (20.0–24.00)	21.0 (18.0–24.0)

Values expressed as mean (SD), median (IQR), or number of patients (%).

*15WT* 15 words test, *CES-D* Center for Epidemiologic Studies Depression Scale, *HADS-A* Hospital Anxiety and Depression Scale-Anxiety Scale, *CAT* COPD assessment test, *EQ5D* EuroQuol 5D, *VAS* Visual Analogue Scale, *SF-12* 12-Item Short Form Health Survey

^*^p ≤ 0.05 vs. group COPD patients

### Social status

Subjects with COPD less frequently had a partner and, when having a partner, they were less likely to be ‘very satisfied’ with the daily support they received from their partner than non-COPD subjects. Subjects with COPD also perceived emotional support less often compared to non-COPD subjects (Table 4). Marital status, personal network size, instrumental support, loneliness, receiving help and employment status were comparable between groups.

**Table 4:** Social status of the study subjects divided in patients with and without COPD

	Non-COPD subjects	COPD subjects
Partner, yes (%)	622 (83.8)^*^	47 (69.1)
Married, yes (%)	535 (72.1)	45 (66.2)
Daily support partner		
No partner/no answer, *n* (%)	118 (18.3)^*^	22 (32.4)
Very dissatisfied, *n* (%)	4 (0.5)	1 (1.5)
Dissatisfied, *n* (%)	8 (1.1)	1 (1.5)
A little dissatisfied, *n* (%)	39 (5.3)	5 (7.4)
Satisfied, *n* (%)	330 (44.5)	26 (38.2)
Very satisfied, *n* (%)	190 (27.2)^*^	10 (14.7)
Personal network size, number	19.0 (13.0–28.0)	16.0 (11.0–25.5)
Instrumental support		
No support, *n* (%)	90 (12.1)	10 (14.7)
Seldom, *n* (%)	400 (53.9)	35 (51.5)
Sometimes, *n* (%)	231 (31.2)	21 (30.9)
Often, *n* (%)	21 (2.8)	2 (2.9)
Emotional support		
No support, *n* (%)	18 (2.4)	4 (5.9)
Seldom, *n* (%)	94 (12.7)	10 (14.7)
Sometimes, *n* (%)	360 (48.5)	38 (55.9)
Often, *n* (%)	270 (36.4)^*^	16 (23.5)
Loneliness		
Emotional, yes (%)	222 (29.9)	25 (36.8)
Social, yes (%)	277 (37.3)	25 (36.8)
General, yes (%)	353 (47.6)	33 (48.5)
Help		
Personal, yes (%)	9 (1.2)	0 (0.0)
Domestic, yes (%)	128 (17.3)	12 (17.6)
Nursing, yes (%)	9 (1.2)	1 (1.5)
Having a paid job, *n* (%)	478 (64.4)	37 (54.4)

Values expressed as mean (SD), median (IQR) or number of patients (%)

*p ≤ 0.05 vs. group COPD patients

## Discussion

This study aimed to investigate the physical, mental and social status of subjects with COPD in a population-based sample in the Netherlands. Despite a mild-to-moderate degree of airflow limitation, subjects with COPD had significant impairments in specific measures of physical, mental and social status compared to non-COPD subjects.

The impact of COPD on physical, mental and/or social status has been demonstrated in multiple studies, mostly including subjects with moderate to very severe COPD recruited at outpatient clinics.^14,16,18–20^ However, this may limit the external validity of these findings toward subjects living with COPD in the general population. The current study shows that COPD subjects with a mean age of 60 years and a mild-to-moderate degree of airflow limitation have a deteriorated physical, mental, and social status when compared to non-COPD subjects. The fact that the subjects with and without COPD had a comparable age, gender distribution, BMI and an equal number of self-reported comorbidities, suggests that the presence of mild-to-moderate COPD may be the main driver of these impairments.

### Physical, mental, and social impact of COPD

Corresponding to the current study, the HELP-COPD study suggested that physical, psychological, social support should be offered from mild disease, routinely providing a holistic approach throughout the life-long course of the disease.^21^ Thus, holistic support should not be left until severe disease when the burden of disease has already become disabling.

Prior research regarding the physical impact of COPD found a high proportion of COPD subjects experiencing pain and incontinence^22,23^ as well as reduced physical performance when compared to non-COPD subjects.^9,11^ The present study also showed that subjects with mild to moderate COPD have a slower walking speed and report lower values on self-rated health surveys compared to non-COPD subjects. Slow walking speed has been associated with disability and increased risk of hospitalization and mortality in patients with moderate to severe COPD.^24^ On the other hand, physical measurements, like sedentary behavior, handgrip strength, number of falls or use of daily aids were comparable. Though, we should take into account that only a categorization was made in disease severity based on FEV_1_% predicted, which is poorly associated with the degree of lung emphysema.^25^ The question remains which factors cause these specific impairments in physical status, while other physical parameters were comparable between subjects with and without COPD. Differences in self-rated health between subjects with and without COPD could be explained by influences of subjective/self-perceived measurement of physical status (e.g., personal fulfillment or expectations).^26^ It can also be hypothesized that subjects already experience dyspnea and/or fatigue during daily activities at an early stage of the disease.^27^ Soumagne and colleagues indicate that lower physical activity can be caused by the nature of symptom development and adaptations to minimize dyspnea provocation.^28^ These questions need to be addressed in future studies. Nevertheless, subjects with a relatively preserved lung function, diagnosed with COPD, experience physical symptoms. This is supported by previous research, indicating that patients with preclinical COPD are physically more inactive than smoking non-COPD peers.^9^ Also, there is growing evidence of significant respiratory morbidity in smokers with preserved spirometry.^29^

With regard to mental status, a more impaired disease-specific health status was observed in subjects with COPD and they rated their health worse than non-COPD subjects, which is in line with previous literature.^6,14^ However, it should be taken into account that the instruments used to assess health status (CAT, EQ5D, and VAS) largely focus on the patients’ personal perspective on bodily sensations and limitations in daily living. These measurements direct less attention to emotional consequences, accentuating the influence of daily limitations and need for adequate management of COPD. No significant differences in cognitive function and mood status were observed between the two groups. In contrast, previous literature showed that subjects with COPD have more symptoms of anxiety and depression and more cognitive dysfunction compared to non-COPD controls.^13,19^ Contradictory results can be explained by the fact that studies were performed with a more severe COPD population, not representing the general COPD population. Overall, the current results indicate that the largest proportion of subjects with COPD from a general population barely experience any mental symptoms.

New insights were gained relating to social consequences of COPD. Subjects with COPD less frequently had a partner, rated daily support from their partner less positively and did not receive emotional support as often as non-COPD subjects. As the current study showed no differences in personal network size and feelings of loneliness between subjects with and without COPD, it appears that the shortage of emotional support does not directly depend on the number of people in their network. This leads to the assumption that subjects with COPD have a higher need for emotional support, which was also found in previous research.^30^ Reduced support may be explained by the fact that subjects with COPD and their partner or caregiver are confronted with multiple limitations in daily living and often have different perceptions on the disease.^31^ Another study showed that, in order to cope with the disease, partners of COPD subjects are very important for the patient.^32^

### Possible consequences for clinical practice

Healthcare professionals should be aware of the fact that subjects with mild COPD in the general population may experience specific impairments in physical, mental and social status. This needs to be taken into consideration when setting up COPD management programs and/or programs to monitor disease progression. Concerning physical status, this can be applied by giving education about the influence of COPD on physical status or provide physiotherapy/physical activity coaching even at an early stage of the disease.^33^ Results with regard to mental status imply that a large proportion of COPD subjects do not experience psychological symptoms. This may suggest that these subjects would sufficiently benefit from a management program with a lower intensity, and presumably, lower healthcare costs.^34^ Assessment of physical, mental and social status at the start of treatment may identify these subjects. However, the cost-effectiveness remains to be established. Early assessment would also provide the opportunity to identify COPD subjects with higher needs for social support and involve close family, friends, and other informal caregivers (i.e., a patient’s social system) in COPD care programs. It was recently shown that unhealthy lifestyle and morbidities are common in resident relatives of COPD patients.^35^ Whether and to what extent these suggestions positively influence physical, mental, or social status of patients remains to be determined.

The present Dutch healthcare guidelines provide comprehensive directives about COPD management implemented by general practitioners, i.e., focusing on airflow limitation, general deterioration, symptoms, and health status.^36^ The results of the current study support these guidelines, as airflow limitation and specific measures of physical and mental status differed significantly between subjects with and without COPD. However, healthcare is not provided to a large proportion of undiagnosed COPD subjects. Since the current study showed that COPD has an impact on specific aspects of all three areas (physical, mental, and social), the necessity of detecting COPD in the general population is emphasized. In the current healthcare system, detection of COPD mainly consists of performing post-bronchodilator spirometry.^37^ Other studies suggest that we should, for example, increase awareness of COPD in smokers^38^ or apply specifically developed questionnaires assessing symptoms^39^ to uncover COPD in the general population. Based on the current results, it is recommended that, in addition to the current guidelines, assessment of functional and health status in subjects with an increased risk for COPD (e.g., a high number of pack-years/current smokers) be made.Instruments have to be quick and easy to implement into clinical practice.^40,41^ Assessment of functional status and health status should be performed for diagnostic purposes and assess the impact of COPD.

### Limitations

A limitation of the current study is that a relatively small number of subjects with COPD participated in the current study. This could be anticipated based on previously published prevalence rates of COPD in a general population^42^ and probably was even more pronounced as the current population was slightly younger than populations examined in previous studies including the Burden of Obstructive Lung Disease (BOLD) study.^43^ Although Longitudinal Aging Study Amsterdam (LASA) is considered a nationally representative sample for the included age range,^44^ results may not be generalized to younger or older age groups. Third, a history of respiratory diseases was defined as self-reported chronic bronchitis, asthma, emphysema, or COPD despite the fact that obstructive lung diseases are frequently underpresented to physicians and underdiagnosed.^45^ It is well recognized that several comorbidities including cardiovascular and peripheral artery disease are more common in COPD than in non-COPD subjects,^46,47^ but no statistically significant differences were shown in the present study. This is probably due to the limited number of patients. Finally, measurements were conducted cross-sectionally, not providing the possibility to determine the causal direction between the physical, mental, and social impact and COPD. It is possible for the causal direction to go the other way, where symptoms are prior to COPD. However, previous longitudinal research suspects otherwise.^48^

## Conclusion

Subjects with mild-to-moderate COPD identified in a general population sample in the Netherlands showed some specific impairments in measures of physical, mental and social status compared to non-COPD subjects. The observations of this study support the use of instruments to assess integrated health status early in the course of the disease and the importance of managing mild-to-moderate COPD patients beyond the degree of lung function impairment.

## Methods

Current data are part of the LASA, an ongoing longitudinal study designed to determine predictors and consequences of physical, emotional, cognitive and social functioning. LASA is based on a nationally representative sample of older adults from three

regions in the Netherlands.^44^ Ethical approval for the LASA study was given by the Medical Ethics Committee of the VU University Medical Center Amsterdam (METC number 2012/361). Extended information of the LASA study design has been published.^44^ Methods were performed in accordance with relevant regulations and guidelines.

### Participants

Subjects aged between 55 and 65 years were recruited between November 2012 and November 2013. Subjects were randomly sampled from 11 municipality registers in the regions Amsterdam, Zwolle, and Oss in the Netherlands. They received information about the study by letter, were contacted by phone and, when approved, subsequently interviewed in their home environment. Other than age, no inclusion or exclusion criteria were defined. All subjects gave written informed consent before entering the study. Only data from subjects who completed the post-bronchodilator spirometry were included in the current analysis. The details about recruitment and assessment have been described in a published protocol.^49^

### Spirometry

Subjects were questioned if they had chronic bronchitis, emphysema, or COPD. Post-bronchodilator spirometry (forced expiratory volume in the first second, FEV_1_, and forced vital capacity, FVC) was performed to assess COPD. Spirometry was conducted with a Vmax Vyntus SPIRO—USB PC Spirometer from CareFusion (Höchberg, Germany), 15 min after inhalation of 200 μg salbutamol. The following criteria for acceptable spirometry were applied: sufficient number (≥3) of successful maneuvers, two highest FEV_1_ or FVC values being within 150 ml, a (maximal volume) exhalation time of at least 6 s and volume–time curve showing no change in volume (<0.025 L) for ≥1 s.^50^ In order to avoid misdiagnosis in this elderly population, COPD was defined according to the lower limit of normal (LLN) instead of the fixed ratio.^51^ Subjects with COPD were categorized in traditional GOLD grades for airflow limitation; grade 1 (FEV_1_ > 80% predicted), grade 2 (FEV_1_ 50–80% predicted), grade 3 (FEV_1_ 30–50% predicted) or grade 4 (FEV_1_ < 30% predicted).^1^

### Assessment of integrated health status

Demographics, smoking history, blood pressure, body mass index (BMI), self-reported comorbid diseases (heart diseases, peripheral artery diseases, diabetes, cerebrovascular disease incontinence, osteoarthritis, rheumatoid arthritis, cancer, and other chronic diseases) and medical history were recorded.

Physical status was assessed by: self-reported health, number of falls in the past year, the experience of pain, sleep quality, self-reported sedentary behavior, handgrip strength, walking 6 m (maximal pace, including 180° turn after 3 m), and use of aids during daily life. Mental status was measured as: cognition using the 15 Words Test (15WT)^52^ overall life satisfaction, mood status was assessed using the Hospital Anxiety and Depression Scale-Anxiety subscale (HADS-A)^53^ and the Center for Epidemiologic Studies Depression Scale (CES-D),^54^ disease-specific health status was assessed using the COPD assessment test (CAT)^55^ generic health status was assessed using EuroQuol 5D and its Visual Analogue Scale (EQ5D VAS)^56^ as well as the 12-Item Short Form Health Survey (SF-12). Social status was assessed as the interviewer asking questions about whether or not the subject had a partner, marital status, daily support from the partner, personal network size, instrumental and emotional support, loneliness (measured using the De Jong Gierveld loneliness scale,^57^ domestic help or help with self-care, and employment.^49^

### Statistics

Subjects with and without COPD were compared. Variables were tested for normality with a Skewness and Kurtosis test. Mean and standard deviation (SD), median and interquartile range (IQR), and/or proportions were used as appropriate. Categorical variables were described as absolute numbers and frequencies. A Mann–Whitney *U* test and one-way analysis of variances (ANOVA) were applied for normally distributed variables. Where appropriate, post hoc least significant difference (LSD) multiple comparisons were performed. A Kruskal–Wallis test was assessed for non-normally distributed variables and a Chi-square test was applied for categorical variables. A *p*-value of less than or equal to 0.05 was considered statistically significant. Statistics were performed with SPSS V.20.0.

### Data availability

LASA data are available for research. To obtain data, researchers need to submit an analysis proposal that is evaluated by the LASA Steering Group. The LASA Steering Group has adopted a policy of open sharing of data with interested researchers for specific research questions on aging-related issues. More information on data requests can be found at the study website: www.lasa-vu.nl.

## Electronic supplementary material

Supplementary file

## Electronic supplementary material

**Supplementary information** accompanies the paper on the *npj Primary Care Respiratory Medicine* website (10.1038/s41533-018-0097-3).
