# Supplementary material for: The physical, mental, and social impact of COPD in a population-based sample: results from the Longitudinal Aging Study Amsterdam
Source: NPJ Prim Care Respir Med. 2018 Aug 10;28:30. doi: 10.1038/s41533-018-0097-3 (PMC6086825; doi:10.1038/s41533-018-0097-3)
Supplement: Supplementary file 1 — Supplementary file [file 41533_2018_97_MOESM1_ESM.docx]

**Title:**

How to determine an impaired health status in COPD? Results from a population-based study

**Authors:**

Dionne E. Smid, Martijn A. Spruit, Dorly J.H. Deeg, Martijn Huisman, Jan Poppelaars,

Emiel F. M. Wouters and Frits M.E. Franssen

**Appendix**

**Figure E1. Flow diagram of subject inclusion**

Total number LASA cohort:

n= 1023

n=121 Participant refused medical interview

n=7 Not contacted for medical interview

n=5 Ineligible

n=1 Decreased

Total number including medical interview:

n= 889

n=18 No spirometry due to physical reasons

n=15 Spirometer did not work/parts missing

n=6 Participant refused spirometry

n=6 No medication available to perform spirometry

n=5 Limited time, unable to perform spirometry

n=4 Environment not suitable to perform spirometry

n=1 Demographic data missing, unable to calculate reference values

n=24 Unknown

Total number of patients with required data:

n= 810
